# CD73 a novel marker for the diagnosis of benign and malignant salivary gland tumors

**DOI:** 10.4317/jced.54918

**Published:** 2019-03-01

**Authors:** Mohammad-Ali Ranjbar, Zahra Ranjbar, Maryam Zahed, Negar Nikookar

**Affiliations:** 1 Department of Oral and Maxillofacial Pathology, School of Dentistry, Shiraz University of Medical Sciences, Shiraz, Iran; 2 Oral and Dental Disease Research Center, Department of Oral and Maxillofacial Medicine, School of Dentistry, Shiraz University of Medical Sciences, Shiraz, Iran; 3 Undergraduate Student, School of Dentistry, Shiraz University of Medical Sciences, Shiraz, Iran

## Abstract

**Background:**

Ecto-5’-nucleotidase (CD73) plays an important role in the development of several types of cancer; however, its prognostic significance in salivary gland tumors remains unknown. The current study was conducted to investigate the expression of CD73 in such tumors.

**Material and Methods:**

In this retrospective study, immunohistochemical expression of CD73 was evaluated in 25 pleomorphic adenomas, 20 mucoepidermoid carcinomas and 20 adenoid cystic carcinomas using the Envision technique. Labeling indices of CD73 expression were calculated and compared between lesions.

**Results:**

Immunohistochemical analysis demonstrated that the CD73 expression was significantly higher in salivary gland tumors than in normal salivary gland tissue (*p*<0.001). CD73 expression was significantly higher in mucoepidermoid carcinoma and adenoid cystic carcinoma compared to pleomorphic adenoma *p*<0.001). In addition, the expression of CD73 was significantly higher in lymph node metastasizing cancers compared to non-metastasizing malignancies (*P*<0.001). In contrast, there was no significant association between CD73 expression and other clinicopathological variables such as age, gender, tumor size and distant metastasis (*P*>0.05).

**Conclusions:**

The findings suggest that CD73 can be an independent and useful biomarker for predicting the clinical behavior of salivary gland tumors.

** Key words:**Ecto-5’-nucleotidase, immunohistochemistry, salivary gland tumors.

## Introduction

Ecto-5’-nucleotidase (ecto-5’-NT; CD73) is a glycosyl phosphatidylinositol-anchored cell surface protein which converts extracellular 5’-AMP to adenosine by dephosphorylation of AMP (1). CD73 plays various crucial roles in physiological and pathophysiological processes, including inflammation (2), hepatic fibrosis (3), renal function (4), myocardial ischemia (5), hypoxia, vascular permeability (6) and platelet function (7).

In addition to its serving as an enzyme, CD73 has been found to be over expressed in several types of human and mouse cancers such as breast (8), gastric (9), pancreatic (10), ovarian (11), melanoma (12) and bladder malignancy (13). The clinical significance of this ubiquitous protein and its association with outcome and prognosis of cancers has already been demonstrated (14). Recently, some studies have revealed that CD73 is a key regulatory molecule of tumor cells and is upregulated in certain malignancies; therefore, it has been suggested that the expression of CD73 is associated with tumor growth, angiogenesis, invasion, metastasis and other characteristics of cancerous tissues (15-25), but the mechanisms involved in such associations have not yet been determined.

Although the expression of this protein has been demonstrated in many types of cancer, little research has been conducted on the prognostic and diagnostic value of CD73 in oral and maxillofacial tumors to date.

Ren *et al.* evaluated the immunohistochemical expression of CD73 in oral squamous cell carcinoma (OSCC) and showed the association of this marker with clinicopathological characteristics of such patients, and suggested that CD73 was a potential prognostic marker for OSCC (16). However, there are no reports available regarding the predictive ability of CD73 expression in tumor cells in patients with salivary gland tumors.

Salivary gland neoplasms comprise approximately 5% of head and neck tumors (27). The diagnosis of such rare pathologies is often not straightforward. The overlapping features of different types of salivary gland tumors may present a diagnostic challenge for oral pathologists and surgeons (28). The most common benign salivary gland tumor is pleomorphic adenoma; and mucoepidermoid carcinoma and adenoid cystic carcinoma are the most prevalent malignant tumors (29). Assessment of salivary gland neoplasms using histopathological evaluation by hematoxylin-eosin (H&E) staining is sometimes difficult. Therefore, for definite diagnosis, in addition to H&E staining, other molecular technique such as immunohistochemistry (IHC) is advised to differentiate these similar tumors (30).

Therefore, the current study aimed to evaluate the expression and prognostic significance of CD73 in common salivary gland tumors and also investigate the value of this protein for diagnosis and prediction of tumor behavior.

## Material and Methods

-Patients

A total of 65 cases of primary salivary gland tumor were selected from the existing records in the pathology laboratory of Khalili Hospital, Shiraz University of Medical Sciences. There were 20 cases of mucoepidermoid carcinoma (MEC), 20 cases of adenoid cystic carcinoma (Adcc), and 25 cases of pleomorphic adenoma (PA). Re-evaluation of H&E-stained sections was performed to confirm the diagnosis and then the cases with adequate cellular tissue were selected for IHC evaluation. The control group consisted of 55 cases with normal salivary gland tissue. Clinical information including the age and gender of the patients as well as the location of the tumors was collected from the patient’s medical files.

-Immunohistochemical method

Paraffin-embedded material was cut into 4μm-thick sections and mounted on poly-L-lysine- coated slides. The sections were deparaffinized with xylen, rehydrated in graded alcohols and washed in distilled water. Antigen retrieval was performed by the application of pepsin enzyme at 35-40°C for 12 minutes. Endogenous peroxidase activity was blocked following a 5-minute incubation period with 3% hydrogen peroxide. Then, the sections were incubated with anti-CD37 antibody (1:100 dilution; abcam (ab91086), Cambridge, MA, USA) for 30 minutes. 3, 3 diamino benzidine (DAB liquid, DAKO Corporation, Denmark) was used as chromogen. Lung carcinoma tissues were used as positive control whilst omission of the primary antibody was considered as negative control.

The slides were assessed under a light microscope (Olympus CX31; Tokyo, Japan) at 400× magnification. CD73 staining was analyzed according to the percentage and intensity of stained cells. For this purpose, 1000 epithelial cells were counted in 5 different fields at 40× magnification. Labeling index (LI) was calculated based on the number of immunostained cells to represent the percentage of CD73 immunopositive cells. In addition, intensity of staining was classified into four different categories consisting of: 0 (lack of staining), 1 (weak staining), 2 (moderate staining) and 3 (intense staining).

-Statistical analysis

The results were analyzed using SPSS version 20.0. Mann-Whitney test, Kruskal Wallis test, Chi-Square test, Spearman´s correlation coefficient and receiver operating characteristic (ROC) curve test were used to compare the results between the groups and to find the association with clinicopathological features such as age, gender, tumor size, tumor grade, metastasis to lymph nodes and distant metastasis. Differences were considered statistically significant at *p*<0.05.

## Results

The demographic data of the patients are shown in  Table 1. The age range of the patients was 27-72 years. Immunohistochemical analysis showed that in the salivary gland tumors 63 (97%) of 65 cases were CD73-positive, while in the control group, 2 (3.6%) of 55 cases were CD73-positive. Therefore, salivary gland tumors revealed significantly higher expression of CD73 than normal salivary gland tissue (*P* < 0.001). Immunoreactivity was detected in the cytomembrane of epithelial cells. Moreover, this marker exhibited a partial positive staining in the cytoplasm.

**Table 1:**
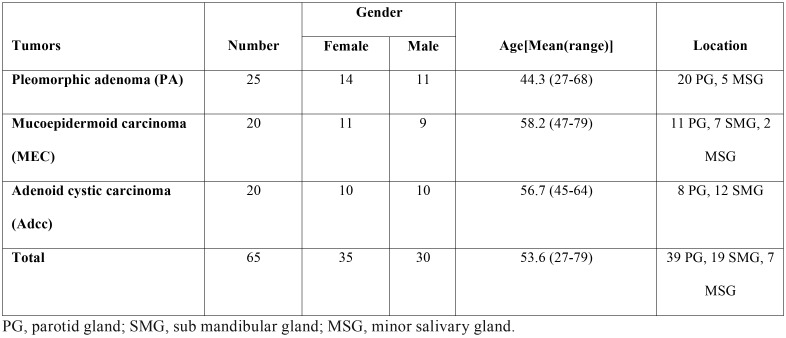
Average age and gender of patients and location of salivary gland tumor.

-Mucoepidermoid Carcinoma

**Figure 1:**
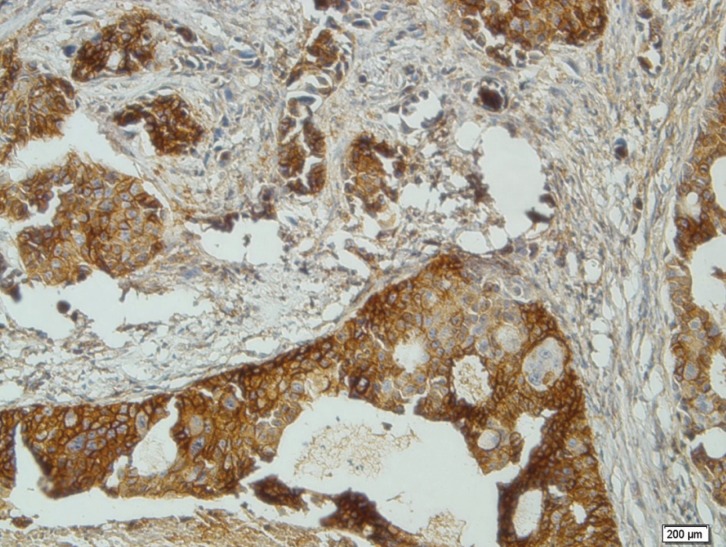
Cytoplasmic and membranous expression of CD73 in Mucoepidermoid carcinoma (×200).

All cases of MEC (including 4 low-grade, 10 intermediate-grade and 6 high-grade tumors) exhibited immunoreactivity of CD73 and showed staining in the squamous and some of the intermediate cell components (Fig. 1). The LI ranged from 40%-80%. There was a significant difference between LI of low-grade MECs (mean, 45± 16.8), intermediate-grade MECs (46.5±16.33) and high-grade MECs (65±17.3) (*p* =0.04).

-Adenoid Cystic Carcinoma

**Figure 2:**
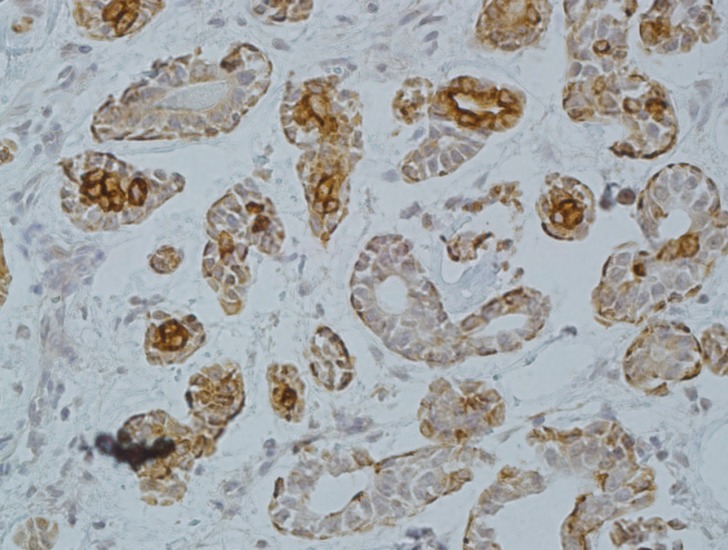
Cytoplasmic and membranous expression of CD73 in Adenoid cystic carcinoma (×200).

The tumors of Adcc consisted of 2 cases of the tubular subtype, 5 cases of the solid subtype and 13 cases of the cribriform subtype. All cases of Adcc were positive for CD73. The range of CD73 LI was 30-70%. The stain was seen in both ductal and myoepithelial components (Fig. 2). The difference in LI between solid (mean: 60±14.4), cribriform (mean: 40.63±11.7) and tubular (mean: 34±10.83) variants of Adcc was not statistically significant (*P*=0.1).

-Pleomorphic Adenoma

**Figure 3:**
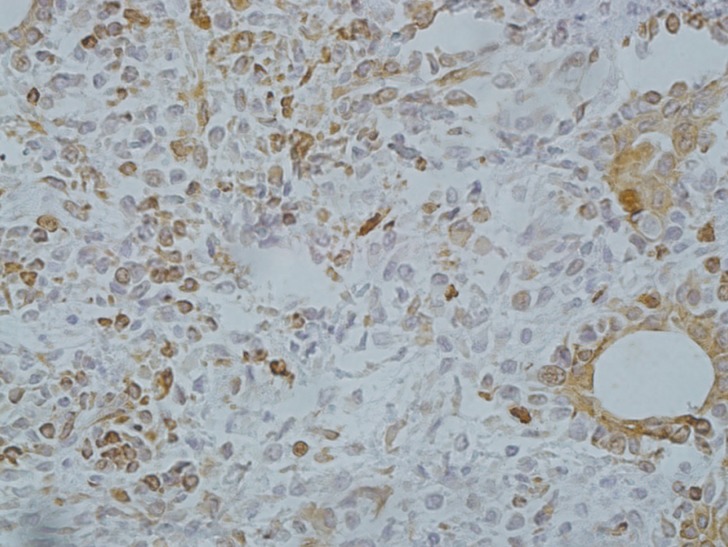
Cytoplasmic and membranous expression of CD73 in pleomorphic adenoma (×200).

Out of 25 cases of PA, 23 demonstrated immunoreaction to CD73. The immunoreactivity was seen in both epithelial and myoepithelial components (Fig. 3). The LI ranged from 0-20%.

The difference in the expression of CD73 between malignant and benign salivary gland tumors was also examined. The LI of CD73 was significantly lower in PA compared to MEC and AdCC (*p*<0.001) ( Table 2). In addition, the difference of intensity of CD73 staining was statistically significant between malignant and benign tumors (*p*<0.001) ( Table 3). No significant difference in CD73 LI was seen between the malignant tumors, but the overexpression of CD73 increased in MEC compared to Adcc (*p*=0.11). Statistically, the expression of CD73 was significantly higher in samples with lymph node metastasis compared with those without metastasis (*P*<0.001). In contrast, there was no significant association between CD73 expression and other clinicopathological variables such as age, gender, tumor size and distant metastasis (*P*>0.05). ( Table 4) According to the receiver operating characteristic (ROC) curve, we obtained a cut-off point of 22.5 % for CD73 (sensitivity: 97.3%, specificity: 100%) to discern between benign and malignant salivary gland tumors.

**Table 2:**
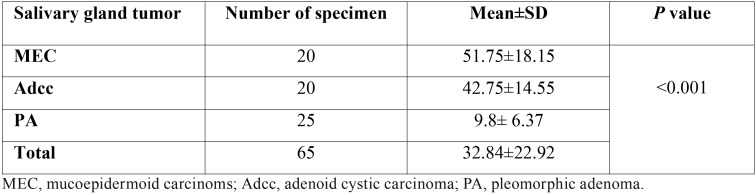
Labeling index (LI) of CD73 in the three types of salivary gland tumors.

**Table 3:**
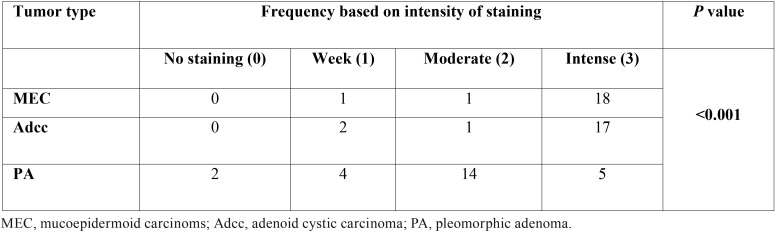
Intensity of staining in the three types of salivary gland tumors (n = 65).

**Table 4:**
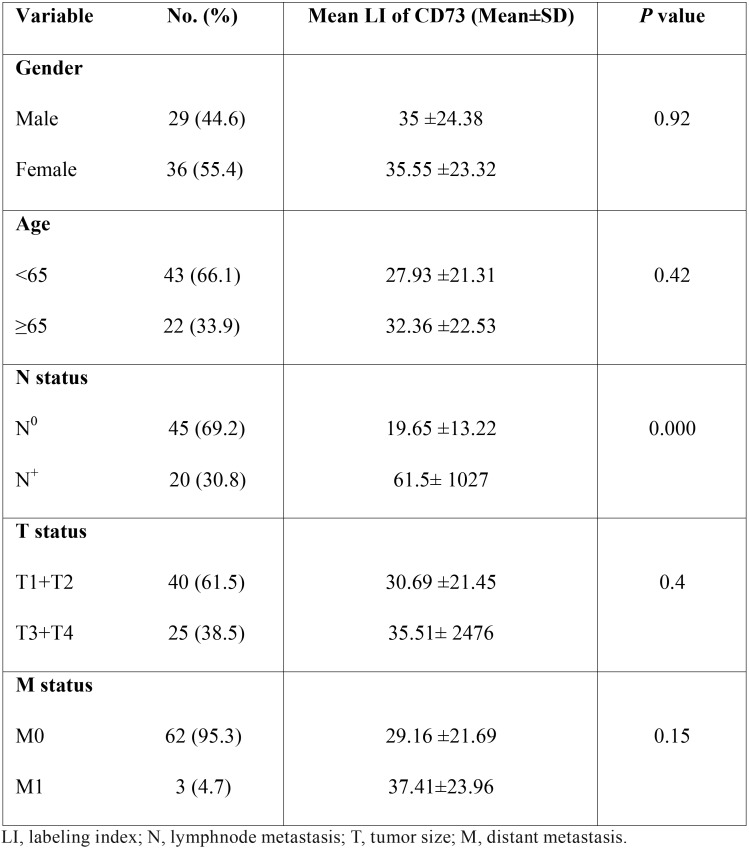
Association between clinicopathological characteristics and CD73 expression in patients with salivary gland tumors.

## Discussion

Current evidence reveals that IHC plays a crucial role in the diagnosis of salivary gland tumors; this important but limited method should be carried out after detailed examination for cancer using H&E staining. Indeed, IHC can be performed to facilitate and support the histiopathological evaluation for definitive diagnosis (30).

To the best of our knowledge, the current work is the first study to evaluate CD73 immunoexpression in benign and malignant salivary gland tumors. The present study clearly showed higher expression of CD73 in salivary gland tumors than normal salivary gland tissue. This result confirms that CD73 is a key regulatory molecule in the tumorogenesis process of these tumors. In addition, the present results revealed that CD73 expression was significantly higher in malignant salivary gland tumors than benign salivary gland tumors (*p*=0.000).

The available findings on CD73 immunoreactivity in other tumors support our findings. Association of high expression of CD73 with poor prognosis of colorectal cancers was reported previously, and the biological properties of CD73 for identification of patients with progressive tumors were demonstrated (15,16). Additionally, another study investigated the expression of CD73 in patients with adenocarcinoma of gallbladder and revealed that CD73 overexpression was associated with tumor progression and survival time of patients. That study also showed that CD73 was an independent marker for the prognosis and clinical behaviors of gallbladder adenocarcinoma (19).

Furthermore, we found that the CD73 expression was higher in lymph node metastasizing salivary gland cancers than non-metastasizing ones. In agreement with our finding, some studies have demonstrated that expression of CD73 may be associated with tumor promotion and this enhances the metastatic feature of cancer cells (31,32). These retrospective studies exhibited that CD73 contributed to metastasis of prostate cancer, gastric cancer and malignant melanoma (12,18,23).

In contrast, there are a number of reports regarding the correlation of CD73 with improved clinical outcome in patients with different solid tumors. A study evaluating the biological role of CD73 in breast cancer showed that increased expression of CD73 could serve as a potential diagnostic marker for breast carcinoma with good prognosis (24). Another study considering the expression of CD73 in epithelial ovarian carcinoma showed higher expression of CD73 in patients with favorable prognosis. Associations of CD73 overexpression with better prognosis, lower stage and better differentiation were demonstrated in this study (17).

However, there are other investigations in agreement with our observations regarding CD73 expression in prostate, gastric and certain types of breast cancer (18,23,25). Inconsistent results considering the role of CD73 in different solid tumors could be explained by the fact that different tissues have various enzymatic activity of CD73 (33).

The present work was a retrospective study with a small sample size. Therefore, randomized prospective studies with a larger sample size should be conducted to support our results. Moreover, other molecular studies are needed to support the idea that CD73 is a prognostic and diagnostic marker in salivary gland tumors.

## Conclusions

In the present study, overexpression of CD73 was observed in malignant salivary gland tumors. Therefore, in addition to the known prognostic parameters, CD73 may serve as a potential biological marker to differentiate malignant and benign salivary gland tumors. Moreover, based on the findings of this study, there was a positive relation between the expression of CD73 and lymph node metastasis. Therefore, immunohistochemical analysis of CD73 may help clinicians to predict the biological behavior of salivary gland malignancies.
